# Moderating effects of individual factors on the relationship between inflammation and psychophysiological states in healthy adults

**DOI:** 10.1016/j.bbih.2025.101135

**Published:** 2025-11-07

**Authors:** Kao Yamaoka, Yuri Ishii, Yuri Terasawa

**Affiliations:** a FANCL Corporation, Research Institute, 12-13 Kamishinano, Totsuka-ku, Yokohama, Kanagawa, 244-0806, Japan; b Department of Psychology, Keio University, 4-1-1 Hiyoshi, Kohoku-ku, Yokohama-shi, Kanagawa, 223-8521, Japan

**Keywords:** Inflammation, Emotion regulation, Sleep quality, Interoceptive awareness, Psychoneuroimmunology, Individual factors

## Abstract

Systemic inflammation affects psychological processes. Although the association between inflammation and psychophysiological state has been extensively investigated in patients with depression or inflammatory disease, how this relationship manifests in healthy individuals is not clearly known. Not all individuals exhibit distress in response to elevated inflammatory markers, suggesting the presence of psychological moderators. Elucidating the effect of elevated inflammatory markers on healthy adults would broaden the understanding of the relationship between inflammation and psychophysiological state. We investigated the moderating effect of individual factors, including emotion regulation, sleep quality, and interoceptive awareness, on the relationship between inflammatory markers and psychophysiological states in healthy adults. A total of 155 participants aged 30–59 years were assessed for inflammatory markers, individual factors, and subjective psychological and physical symptoms. Hierarchical regression and interaction models revealed that individuals with poor emotion regulation or low-quality sleep showed stronger associations between inflammatory markers and symptoms such as fatigue, somatic complaints, depression, and anxiety. Conversely, individuals with effective emotion regulation or high-quality sleep exhibited attenuated or even reversed associations, suggesting protective effects. Interoceptive awareness showed weaker and more context-dependent moderating effects. These results highlight the importance of psychological traits in modulating the effects of inflammation on mental and physical well-being in clinically healthy adults. Targeted interventions for enhancing emotion regulation and sleep quality may mitigate the psycho-physiological burden of inflammation and reduce the risk of future disease onset. The findings underscore the need for individualized psychoneuroimmunological models that incorporate trait-level moderators to explain variability in stress-related health outcomes.

## Highlights

• Systemic inflammation affects psychological processes.
• Impact of individual factors on inflammation-psychophysiological states is unknown.
• Emotion regulation and sleep affect inflammation-psychophysiological association.
• Systemic inflammation interacts dynamically with individual factors.
• Psychophysiological states vary depending on psychological traits.

## 1 Introduction

Depression is increasing and has emerged as a significant global public health concern with profound economic and social consequences. Existing treatment approaches often fail to achieve sufficient therapeutic efficacy, and many individuals experiencing depressive symptoms remain undiagnosed and untreated. Furthermore, only approximately 30–35 % of adult patients receiving standard treatments achieve remission (Alexopoulos, 2005; Andrews et al., 2004; Chisholm et al., 2004; Roose and Schatzberg, 2005). For more than three decades, mainstream pharmacological treatments for depression have largely targeted monoaminergic neurotransmitters, such as serotonin and dopamine (Cipriani et al., 2018; Harmer et al., 2017). However, recently, increasing attention has been directed toward *inflammation* as a novel therapeutic target in depression, suggesting a paradigm shift in our understanding of its pathophysiology (Amasi-Hartoonian et al., 2022; Kohler et al., 2016; Yin et al., 2024). Previous research has shown that infection in early life increased the risk of depression (Goodwin, 2011). More direct evidence of the relationship between inflammation and depression has been demonstrated by an increase in interleukin-6 (IL-6), C-reactive protein (CRP), and tumor necrosis factor-alpha (TNF-α) associated with a higher risk of depression and increased symptoms (Felger, 2018; Haapakoski et al., 2015; Huang et al., 2019; Khandaker et al., 2014; Paganin and Signorini, 2024; Sorri et al., 2017; Stephenson et al., 2024). Other imaging studies have shown that the structural and functional features in the brain, including the amygdala, were associated with depression and other emotional processes in chronically stressed participants (Han and Ham, 2021; O'Connor et al., 2009; Zhang et al., 2023; Zhou et al., 2024). Furthermore, the effect of anti-inflammation on depression in treatment studies indicate a potential role of cytokine modulation in novel drugs for depression, as the anti-inflammatory treatment decreased depression symptoms in numerous previous studies (Kappelmann et al., 2018; Kohler et al., 2014; Osimo et al., 2020; Roman and Irwin, 2020). Collectively, the evidence from these previous studies indicates the importance of inflammation in depression and other emotional processes.

### 1.1 Relationship between inflammation and psychophysiological states: psychological and central nervous system changes leading to inflammation

Increasing literature has emphasized that depression is not solely a disorder of neurotransmitter dysfunction; it is also strongly associated with immune system activity, particularly inflammation (Lamers et al., 2019; Nusslock et al., 2024; Wittenberg et al., 2020). Traditionally, inflammation is a primary physiological response to physical injury or infection. However, recent findings indicate that psychological stress can also trigger inflammation within the body (Kiecolt-Glaser et al., 2005).

In human research, inflammation is typically assessed through fasting blood samples or saliva collection using biomarkers, such as CRP, interleukin-1β (IL-1β), IL-6, and TNF-α, to quantify systemic immune activity. This study evaluated inflammation via blood-derived biomarkers, including CRP, IL-1β, IL-6, and TNF-α. These are well-established indicators of systemic inflammation and reflect the overall activation state of the peripheral immune system (Moriarity et al., 2023; Ng et al., 2018; Osimo et al., 2020; Rhie et al., 2020). Furthermore, these markers are among the most consistently implicated in psychoneuroimmunology. Meta-analytic evidence indicates elevated levels of IL-6, CRP, and TNF-α in individuals with depression (Dowlati et al., 2010; Paganin and Signorini, 2024; Rengasamy et al., 2021). Similarly, increased concentrations of IL-1β, IL-6, and TNF-α have been reported in older adults with depression (Ng et al., 2018) and across different depressive subtypes (Osimo et al., 2020). CRP, in particular, is the most widely used inflammatory marker in clinical practice (Yeh, 2004) and is well characterized in both medical and psychiatric conditions (Danesh et al., 2000; Fernandes et al., 2015). Higher CRP levels frequently precede the onset of depression in population studies (Gimeno et al., 2009; Khandaker et al., 2014), and Mendelian randomization suggests causal links between IL-6, CRP, and depression (Khandaker et al., 2020). Experimental studies also show that IL-6, IL-1β, and TNF-α rise in response to acute psychosocial stressors such as the Trier Social Stress Test, and that IL-6 correlates with negative affect and stress-related anger or anxiety (Carroll et al., 2011; Slavich and Irwin, 2014). Blood sampling was employed as the most reliable approach, as it provides superior sensitivity and reproducibility compared to saliva or urine, which are subject to variability due to circadian rhythms, flow rate, oral health, and confounding by diet, medication, or renal function (Baldan-Martin et al., 2023; Szabo and Slavish, 2021). Hereafter, we refer to systemic inflammation that is associated with these markers as *inflammation*.

### 1.2 Psychological stress, inflammation, and the evolutionary role of the innate immune system

Psychological stress, a major risk factor for depression, provoked inflammatory responses, an effect rooted in the evolutionary development of the innate immune system. Specifically, physiological systems originally evolved to prepare the body for physical confrontation with predators or adversaries were being activated by symbolic, social, and anticipated threats in modern contexts (Slavich and Irwin, 2014). Experimental studies have provided evidence supporting this theory. Laboratory paradigms, such as the Trier Social Stress Test (TSST) and social exclusion tasks (e.g., Cyberball), elicited inflammatory responses associated with increased neural activity in brain regions, including the dorsal anterior cingulate cortex (dACC) and insula, involved in processing social pain (Eisenberger, 2012; Slavich et al., 2010).

From a neurobiological perspective, psychological stress engages higher-order cortical regions, such as the dorsolateral prefrontal cortex (dlPFC), dACC, and insula. These regions communicate with autonomic nuclei in the hypothalamus and brainstem to regulate peripheral inflammatory activity through three key pathways: the hypothalamic–pituitary–adrenal (HPA) axis, sympathetic nervous system, and efferent vagus nerve (Irwin and Cole, 2011). Crucial mediators in this regulatory cascade include glucocorticoids, adrenaline, noradrenaline, and acetylcholine, all playing essential roles in inflammation modulation. Importantly, these systems can become dysregulated under chronic stress. Specifically, prolonged HPA axis activation may lead to glucocorticoid resistance, in which the anti-inflammatory effects of cortisol are blunted, resulting in upregulated production of pro-inflammatory cytokines (Knight et al., 2021). Consequently, sustained stress can perpetuate a maladaptive state of heightened inflammation, thereby increasing vulnerability to stress-related physical and mental health conditions.

### 1.3 Bidirectional links between inflammation and psychophysiological states

Inflammation is a physiological response to physical injury or infection and a significant factor in modulating psychophysiological states. Canonical pro-inflammatory cytokines, such as CRP, IL-1β, IL-6, and TNF-α, influence the central nervous system (CNS) function and contribute to changes in mood (Felger and Lotrich, 2013; Raison et al., 2006), social behavior (Jolink et al., 2022), emotion regulation (Appleton et al., 2013), and the onset and prognosis of depressive disorders (Khandaker et al., 2014; Osimo et al., 2020). Experimental studies revealed that vaccine-induced inflammatory responses in healthy individuals were associated with increased anxiety, depressive symptoms, and social withdrawal (Balter et al., 2018; Jolink et al., 2022, 2024; Kuhlman et al., 2018). These behaviors, often referred to as “sickness behaviors,” hold a significant interest in psychoneuroimmunology (Dantzer et al., 2008).

Mechanistically, cytokines may cross the blood–brain barrier (BBB) or signal the CNS via the vagus nerve, which can influence brain regions critical to emotion, such as the amygdala and prefrontal cortex (PFC) (Harrison et al., 2009; Inagaki et al., 2012). These changes are associated with increased anxiety and diminished attentional control. Patients with major depressive disorder exhibit BBB dysfunction, which leads to immune cell infiltration, glial activation (including microglia and astrocytes), and excess release of pro-inflammatory mediators and reactive oxygen species. This ultimately results in neuroinflammation and neuronal damage (Wu et al., 2021).

### 1.4 Inconsistency and individual differences in the Inflammation–Psychophysiological state

Despite robust evidence linking inflammation to mental and behavioral outcomes, their relationship remains inconsistent. While inflammation can influence psychological functioning, subjective well-being and behavior do not always correspond to the levels of the inflammatory markers. Some individuals with elevated CRP or IL-6 levels report no psycho-physiological distress (Marsland et al., 2006; Udina et al., 2012). This dissociation suggests the presence of individual differences that moderate the effects of inflammation on psycho-physiological functioning. Recent studies highlight that individual factors, such as sex (Lamers et al., 2019), depression severity (Wittenberg et al., 2020), emotional clarity (Stephenson et al., 2024), and social relationships (Jolink et al., 2022, 2024), play a role in shaping these associations. Hence, vulnerability and contextual factors may determine the degree to which inflammation affects mental and physical health.

### 1.5 Candidate moderators: interoception, emotion regulation, and sleep

Among the possible moderators, *interoception*, *emotion regulation*, and *sleep* have gained increasing attention for their roles in linking inflammation to subjective states.

*Interoception* refers to the perception and interpretation of internal bodily signals, such as heart rate, respiration, digestion, and visceral sensations (Craig, 2002), and has been closely associated with emotion recognition and regulation. Accurate perception and integration of bodily changes underpin subjective emotional experiences. Hence, subjective awareness of inflammation may represent an interoceptive process. Functional imaging studies have shown that inflammation is associated with increased activity in interoceptive neural networks, particularly within the anterior insula and ACC (Kraynak et al., 2018; Savitz and Harrison, 2018). Furthermore, this activity was correlated with cytokine levels and perceived sickness (Harrison et al., 2009; Lekander et al., 2016). These findings suggest that interoception may mediate the translation of physiological inflammation into subjective experience.

*Emotion regulation* refers to the monitoring, evaluation, and modification of emotional reactions to achieve one's goals (Gross and John, 2003; John and Gross, 2004). Adaptive regulation strategies, such as cognitive reappraisal, were associated with better mental and physical health outcomes, whereas maladaptive strategies, such as suppression, were linked to heightened depressive symptoms and increased inflammatory activity (Appleton et al., 2013; Moriarity et al., 2023). Longitudinal research revealed that a greater use of reappraisal was associated with reduced IL-6 levels over time (Jones et al., 2023), whereas higher reliance on suppression predicted elevated CRP (Appleton et al., 2013). These findings highlight emotion regulation as a potential pathway through which inflammation influences mental health.

*Sleep* is a complex physiological process regulated by the interaction between homeostatic and circadian mechanisms (Ballesio, 2023). While distinct from interoception and emotion regulation, it is equally essential for maintaining psychophysiological health and immune homeostasis (Frey et al., 2007; Haack et al., 2007; Yoo et al., 2007). Both acute and chronic sleep deprivation impaired cognitive and emotional functioning (Bajaj and Kaur, 2022; Thompson et al., 2022), leading to increased anxiety and deficits in attention and memory (Chee and Chuah, 2007; Tomaso et al., 2021). These impairments were driven by changes in the neuroendocrine and inflammatory systems (Haack et al., 2007; Irwin et al., 2008). Indeed, 24-h sleep deprivation increased IL-6 and CRP levels while reduced cortisol, which suggested dysregulation of the HPA axis as a potential mechanism (Thompson et al., 2022). Moreover, recent studies that examined the triadic relationship between sleep, inflammation, and depression suggested inflammation partially mediated the association between sleep disturbances and depressive symptoms (Yin et al., 2023). These findings reinforce the centrality of sleep in regulating inflammation-related psychological outcomes.

### 1.6 Present study

Evidence highlights a bidirectional and interactive relationship between systemic inflammation and psycho-physiological states, including symptoms of anxiety and depression (Dantzer et al., 2008; Slavich and Irwin, 2014). Although elevated inflammatory markers are generally associated with worsened psychological outcomes or related behaviors, this association is inconsistent across individuals; some exhibit minimal psycho-physiological symptoms despite elevated inflammation levels (Haack et al., 2007). This heterogeneity suggests that individual-level moderators may influence the extent to which inflammation impacts subjective states. Based on prior findings, interoception, emotion regulation, and sleep were identified as strong candidates for individual-level moderators that shape the inflammation–subjective state relationship (Craig, 2002; Thayer and Lane, 2000; Yoo et al., 2007). Dysfunctions in any domain were linked to heightened inflammation and increased vulnerability to psycho-physiological distress (Kraynak et al., 2018; Nusslock et al., 2019; Tamm et al., 2019). However, limited studies have systematically examined how these factors interact with inflammatory processes to shape subjective experiences. This study hypothesized that individuals with poorer interoceptive awareness, impaired emotion regulation, and insufficient sleep would be more vulnerable to the detrimental psychological and physical effects of increased inflammation. Conversely, individuals with greater interoceptive sensitivity, stronger emotion regulation abilities, and better sleep quality would be more resilient to these effects.

To assess this hypothesis, we conducted an exploratory study with a community-based sample of healthy adults across a wide age range. As inflammation sometimes influences somatic symptoms (e.g., fatigue) more strongly than cognitive-affective symptoms (Frank et al., 2021; Fried et al., 2020), we considered both psychological and physical dimensions. To avoid confusion, each moderator was also individually examined to elucidate its unique role.

## 2 Methods

### 2.1 Participants

This study recruited 178 healthy adults (both male and female) between December 2023 and February 2024. Participants were eligible if they were aged 30–59 years, had a body mass index (BMI) of 18.5–30.0 kg/m^2^, and had graduated from high school. Exclusion criteria included a current or past diagnosis of dementia, depression, or other psychiatric disorders, individuals who were pregnant or breastfeeding or could become pregnant during the study period, and those unable to discontinue medications or health supplements that could influence study outcomes (Supplemental Material 1). Furthermore, to minimize the effect of the existing inflammatory conditions, individuals over 59 years old of age, those with a high BMI, current smokers, or those who had quit smoking within 12 months prior to providing informed consent were excluded. This was because aging (Sendama, 2020), high BMI (Cohen et al., 2021; Nieman et al., 1999) and smoking habits (Yanbaeva et al., 2007) can significantly affect levels of inflammation. Further, individuals previously diagnosed with alcohol-related diseases or those being treated for such diseases, individuals with severe current or past medical conditions that require ongoing pharmacological treatment, such as neurological disorders, malignant tumors, immune system diseases, diabetes, liver diseases (e.g., hepatitis), kidney diseases, cardiovascular diseases, thyroid disorders, adrenal disorders, or other metabolic diseases, individuals who have previously had dysregulation of the hypothalamic-pituitary-adrenal axis, individuals with a history of gastrointestinal surgery (e.g., gastric resection), individuals with periodontitis or those being treated for periodontitis, as well as individuals deemed unsuitable as participants by the principal investigator or study physician based on clinical laboratory test results, physical measurements, or physical examination findings were excluded from the study.

**Fig. 1:**
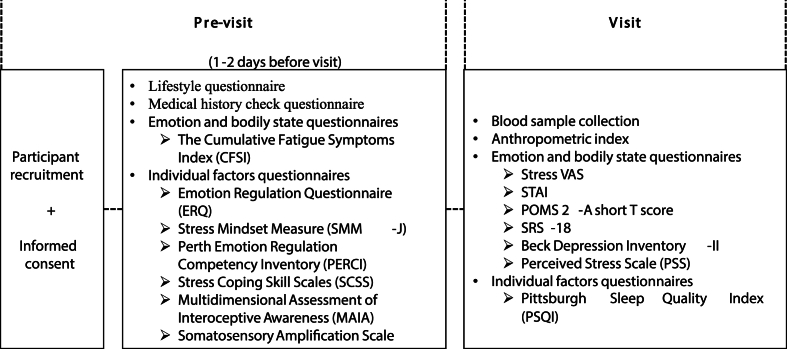
Study timeline and pre-visit and visit day schedule.

All participants provided informed consent online in accordance with the Declaration of Helsinki. Subsequently, 19 individuals were excluded for various reasons, including failure to meet eligibility criteria during the clinic visit, vasovagal reactions during blood collection, and scheduling conflicts. Consequently, 159 participants (89 females; age range: 30–59 years; mean age: 44.7 ± 8.3 years) were included in the initial analyses. After study completion, four participants were excluded due to high BMI (>30.0 kg/m^2^), resulting in a final sample of 155 participants. This study was approved by the Clinical Research Ethics Committee of AMC Nishi-Umeda Clinic (Approval No. RD2023-4) and was pre-registered with the Japanese Conference of Clinical Research (UMIN000053382). Participant recruitment, screening, and clinical assessments were conducted by HUMA R&D CORP (Tokyo, Japan). Fig. 1 illustrates the overall study flow.

### 2.2 Psychological assessments

In this study, the term *individual factors* referred to relatively stable psychological traits or abilities that may modulate responses to stressors. These included cognitive frameworks (e.g., stress mindset), emotional regulation capabilities (e.g., emotion regulation strategies and competencies), sensitivity to internal bodily sensations (e.g., somatosensory amplification, interoceptive awareness), and sleep quality. These factors were primarily assessed using self-report questionnaires that evaluated habitual thought patterns and psychological skills, considered less susceptible to transient situational influences. Japanese versions of the Stress Mindset Measure (SMM-J) (Crum et al., 2013; Iwamoto et al., 2020), Coping Inventory for Stressful Situations (CISS) (Kijima, 2008), Emotion Regulation Questionnaire (ERQ) (Gross and John, 2003; Yoshizu et al., 2013), Perth Emotion Regulation Competency Inventory (PERCI) (Preece et al., 2018; Tsujimoto et al., 2022), Pittsburgh Sleep Quality Index (PSQI) (Buysse et al., 1989; Doi et al., 1998, 2000), Multidimensional Assessment of Interoceptive Awareness (MAIA) (Shoji et al., 2018), and Somatosensory Amplification Scale (Barsky et al., 1990; Nakao et al., 2001) were employed to assess individual factors. Notably, the PSQI assessed sleep quality over the past month, which provided a clear temporal frame. Although sleep is governed by complex physiological mechanisms involving homeostatic processes and circadian rhythms (Ballesio, 2023), it is an integral component of psychological resources due to its close association with emotional states (Yoo et al., 2007) and involvement in inflammation-related depressive symptoms (Yin et al., 2023).

Conversely, *psychophysiological states* refer to the subjective experiences of physical fatigue, emotional responses, perceived stress, and resultant psychological burden encountered in daily life. These states encompassed both psychological aspects (e.g., depression, anxiety, tension, stress) and physical aspects (e.g., fatigue, somatic discomfort), and were characterized by their situational and variable nature. This study used the Japanese versions of Cumulative Fatigue Symptoms Index (CFSI) (Kosugi et al., 1992), Beck Depression Inventory-II (BDI-II) (Beck et al., 1961), Profile of Mood States 2-A Short Form (POMS 2-A Short T Score), State-Trait Anxiety Inventory (STAI) (Spielberger et al., 1983), Stress Visual Analog Scale (VAS), Perceived Stress Scale (PSS) (Iwahashi et al., 2002), and Stress Response Scale-18 (SRS-18) (Suzuki et al., 1997). Notably, the Trait Anxiety subscale of the STAI, which measured a general tendency to perceive situations as threatening, was excluded as it did not align with the definition of situational psycho-physiological states.

Psychological assessments were administered in two phases. After consent was obtained, participants completed lifestyle questionnaires and the Stress Mindset Measure, CISS, ERQ, PERCI, MAIA, and Somatosensory Amplification Scale online 1–2 days prior to their clinic visit. On the day of their clinic visit, participants completed the remaining assessments: the BDI-II, STAI, Stress VAS, PSS, SRS-18, and PSQI. Additionally, questionnaires that assessed recent gastrointestinal symptoms (e.g., constipation, diarrhea) were administered; however, these have not been discussed in this study.

### 2.3 Inflammatory reactivity

Assessed inflammatory biomarkers were CRP, IL-1β, IL-6, and TNF-α. Blood samples were collected during the clinic visit. Approximately 10 mL of blood was drawn via venipuncture and stored at room temperature in EDTA-containing vacutainer tubes for up to 2 h before processing. Plasma samples were analyzed by BML, Inc. (Tokyo, Japan) via highly sensitive assays to ensure accurate quantification of the inflammatory markers. CRP, IL-1β, IL-6, and TNF-α levels were measured via the N Latex CRP II assay (Siemens Healthineers, Erlangen), Quantikine HS Human IL-1β/IL-1F2 Immunoassay (Funakoshi Co., Ltd., Tokyo), Quantikine HS ELISA Human IL-6 kit (Funakoshi Co., Ltd., Tokyo), and Quantikine HS ELISA Human TNF-α Immunoassay (Funakoshi Co., Ltd., Tokyo), respectively. These had a sensitivity of 0.002, 0.2, 0.16, and 0.2 pg/mL, respectively. To address skewed distributions, all biomarker values were natural log-transformed prior to analysis. Inflammatory reactivity was computed based on their log-transformed concentrations.

### 2.4 Statistical analysis

#### 2.4.1 Factor analysis of questionnaire items

Given the complexity and large number of items assessing individual factors, an exploratory factor analysis (EFA) was conducted to reduce dimensionality and enhance the model's precision. Four aggregate scores (PERCI negative total, PERCI positive total, PERCI total, and PSQI global score) from the 36 items were excluded, which resulted in 32 items for analysis. Maximum likelihood estimation with Varimax rotation was employed. The number of factors was determined based on Scree plot inspection and Kaiser-Guttman criterion (eigenvalues >1).

#### 2.4.2 Moderation analysis of individual factors on the relationship between inflammation and psychophysiological states

To examine whether individual factors moderated the relationship between inflammation and psychophysiological states, hierarchical multiple regression analyses were performed. Inflammation markers, psychophysiological states, and individual factors served as independent variables, dependent variables, and moderators, respectively. Given this study's exploratory nature, multiple comparison corrections were not applied; however, effect sizes (β coefficients and 95 % confidence intervals) were reported alongside p-values to facilitate interpretation.

All analyses were controlled for sex, age, and BMI. In Model 1, the main effects of inflammation markers and individual factors were assessed. Model 2 included the interaction terms between inflammation markers and individual factors. Prior to creating the interaction terms, variables were mean-centered to mitigate multicollinearity. Significant interactions were further explored via simple slope analyses at ±1 standard deviation. All statistical analyses were conducted using JMP® version 18.0.0 (SAS Institute Inc., Cary, NC, USA), with a two-tailed significance threshold set at p < .05.

### 2.5 Daily health log

Participants maintained a daily health log from the day they provided informed consent until the day of testing (average 19 days ± 1.44, range 17 days–21 days). The health log captured information on their overall health status, sleep patterns, stressful events, physical activity, dietary intake of prebiotics, probiotics, other fermented foods, and medication usage. The restricted items (see Supplemental Material 2) comprised foods or beverages rich in dietary fiber, flavonoids, vitamins, minerals, polyphenol-containing products, fermented foods, and whole grains—many of which are known to exert anti-inflammatory effects, as reflected in the Empirical Dietary Inflammatory Pattern (EDIP) and anti-inflammatory food groups (Tabung et al., 2016). Conversely, a limited subset of restricted items included refined grains, high–glycemic index foods, high-fat dairy products, and alcoholic beverages, which have been associated with potential pro-inflammatory effects. To ensure transparency, participants were instructed to report any consumption of these foods or beverages in their daily health logs. Participants also provided detailed accounts of stressful events, including perceived stressfulness and its impact on daily functioning.

## 3 Results

**Fig. 2:**
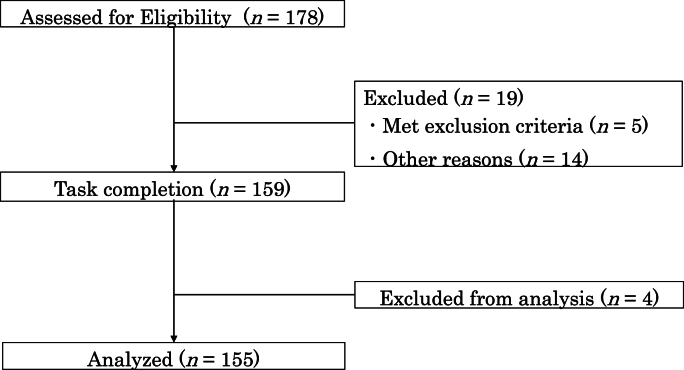
Flow diagram for study participants.

After excluding four participants, data from 155 healthy adults (87 women, 68 men) aged 30**–**59 years (mean age = 44.8, SD = 8.3) were analyzed (Fig. 2). Prior to analysis, a priori power analysis was conducted to determine the minimum sample size required to detect interaction effects. Based on Cohen's (1988) criteria (effect size f^2^ = 0.05, α = .05, power = 0.80), the required sample size was estimated at 158. Thus, 155 participants were deemed sufficient to detect at least moderate-sized effects. Regarding inflammatory biomarkers, CRP, TNF-α, and IL-6 were successfully measured in 97–100 % of samples. However, IL-1β was detected in <1 % of cases and was excluded from all subsequent analyses. Table 1 presents the participants' demographic characteristics**.**

**Table 1:** Participants’ demographic characteristics.

Sample characteristics	*n*	%	*M*	*SD*
Sex				
Male	72	44		
Female	90	56		
Education				
Secondary	23	14		
Undergraduate	126	78		
Post graduate	13	8		
Employment				
Unemployed	10	6		
Employed	141	87		
Self-employed	11	7		
Age			44.78	8.43
BMI			23.84	2.86

### 3.1 Dietary intake and physical activity in relation to inflammation

To examine potential lifestyle effects on inflammation, we first considered dietary deviations. According to the daily health log, a small number of participants reported consuming restricted foods, specifically red wine and beer, although intake levels were within a normative range. Notably, 7 participants consumed beer on more than 50 % of study days. However, a group comparison between these participants and the remaining 148 showed no significant differences in CRP (*p* = .866), IL-6 (*p* = .927), or TNF-α (*p* = .304). In addition, seven participants reported alcohol consumption on the day prior to blood sampling, including two who were frequent beer consumers. Group comparisons between these participants and the remaining 148 revealed no significant differences in inflammatory markers (CRP *p* = .111; IL-6 *p* = .375; TNF-α *p* = .627). These findings suggest that deviations from the dietary restrictions, particularly moderate alcohol consumption, did not exert a measurable influence on the primary outcomes of the present study.

Next, we examined whether physical activity, indexed by total metabolic equivalent of task (MET) values (Ainsworth et al., 2011; Herrmann et al., 2024), was associated with inflammatory markers. Of the 155 participants, 53 engaged in some form of physical activity during the study period. Group comparisons between participants with physical activity (n = 53) and those without (n = 102) revealed no significant differences in CRP (*p* = .128), IL-6 (*p* = .089), or TNF-α (*p* = .714). Moreover, within the physical activity group, MET was not significantly correlated with inflammatory markers (CRP *p* = .929; IL-6 *p* = .476; TNF-α *p* = .894). These results indicate that physical activity did not exert a measurable influence on the primary outcomes of the present study.

### 3.2 Factor analysis of individual psychological traits

To improve the model's clarity and enhance interpretability, EFA was conducted on the questionnaire items that assessed individual psychological traits. The initial EFA yielded eight factors. Sampling adequacy was confirmed via the Kaiser-Meyer-Olkin (KMO) measure, which exceeded 0.80, and Bartlett's test of sphericity, which was statistically significant (*p* < .05). This indicated that factor analysis was appropriate. Six items with low KMO values were subsequently removed, and a revised EFA using maximum likelihood extraction and Varimax rotation was conducted. This re-analysis yielded six factors, with factor retention based on eigenvalues >1 and examination of the Scree plot. Items with factor loadings <0.30 were excluded, which resulted in a final factor solution that explained 65.34 % of the total variance, and loadings ranged from 0.30 to 0.87. This confirmed a well-defined structure that represented the key psychological constructs.

Table 2, Table 3, Table 4 present the results of the factor analysis, including the initial eigenvalues, proportion and cumulative proportion of variance explained by each factor, and the factor correlation matrix, respectively. The first factor was characterized by items such as “I cannot carry out tasks when feeling negative emotions” and “I find negative emotions difficult to accept,” which reflected heightened difficulty in regulating negative emotional states. Therefore, this factor was labeled *Negative Emotion Regulation Difficulty*. The second factor comprised items that reflected bodily awareness and sensitivity to internal physiological cues, such as “I listen to my body to understand how I feel” and “I notice bodily changes when I am angry.” Because of its focus on internal bodily cues, this factor was labeled *Interoceptive Awareness*. The third factor included items related to the difficulty of sustaining or accepting positive emotions, such as “I cannot perform tasks when feeling positive emotions” and “I find positive emotions hard to accept.” These items suggested maladaptive responses even during positive affective states. Hence, this factor was labeled *Positive Emotion Regulation Difficulty*. The fourth factor comprised items related to sleep behavior, such as subjective sleep quality and sleep onset latency over the past month. Since these items reflected variations in sleep-related functioning, the factor was labeled *Sleep Disturbance*. The fifth factor contained items such as “I try to come up with several solutions to a problem,” which represented proactive engagement and planning during stress, and was labeled *Task-Oriented Coping*. Finally, the sixth factor included items that tapped into awareness of neutral, pleasant, or unpleasant bodily sensations, such as “I notice how my body changes when I am angry.” This factor was labeled as *Somatic Awareness*, reflecting the capacity to monitor physiological signals irrespective of their affective valence.

**Table 2:** Results of the factor analysis.

Questionnaire component	Factor loading						
	1	2	3	4	5	6	Communality
Factor 1: Negative emotion regulation difficulty							
Negative-inhibiting behaviour (PERCI)	**0.76**	−0.07	0.10	0.19	−0.08	−0.08	0.65
Negative-tolerating emotions (PERCI)	**0.73**	0.16	0.08	0.12	−0.11	−0.18	0.11
Negative-inhibiting behaviour (PERCI)	**0.73**	−0.02	0.41	0.11	0.13	−0.06	0.49
Emotion-oriented coping (CISS)	**0.70**	0.00	0.13	0.14	−0.02	0.29	0.63
Somatosensory amplification scale	**0.66**	0.10	0.00	0.24	0.19	0.25	0.68
Not worrying (MAIA)	**−0.61**	0.14	−0.03	−0.24	0.08	−0.16	0.53
Negative-controlling experience (PERCI)	**0.60**	−0.30	0.23	0.23	−0.36	0.01	0.77
Not distracting	**−0.31**	0.03	−0.09	−0.02	0.02	−0.04	0.59
Stress mindset measure (SMM-J)	**−0.30**	−0.03	0.25	−0.10	0.15	0.16	0.21
Factor 2: Interoception							
Body listening (MAIA)	0.05	**0.86**	0.04	−0.11	0.02	−0.06	0.68
Emotional awareness (MAIA)	0.11	**0.80**	−0.10	−0.06	−0.05	0.12	0.64
Attention regulation (MAIA)	−0.21	**0.76**	−0.07	−0.09	0.04	0.01	0.73
Trusting (MAIA)	0.01	**0.75**	−0.04	−0.18	0.05	0.00	0.63
Self-regulation (MAIA)	−0.10	**0.68**	−0.06	−0.13	0.16	0.06	0.57
Factor 3: Positive emotion regulation difficulty							
Positive-activating behavior (PERCI)	0.31	−0.14	**0.84**	0.00	−0.05	0.03	0.83
Positive-tolerating emotions (PERCI)	0.04	−0.06	**0.72**	−0.08	−0.27	−0.11	0.61
Positive-inhibiting behavior (PERCI)	0.41	−0.04	**0.65**	0.06	0.12	0.05	0.61
Positive-controlling experience (PERCI)	0.37	−0.32	**0.38**	0.15	−0.36	0.16	0.61
Factor 4: Sleep problem							
Subjective sleep quality (PSQI)	0.09	−0.04	−0.07	**0.87**	0.01	−0.01	0.55
Sleep latency (PSQI)	0.21	0.00	0.04	**0.54**	−0.05	−0.03	0.61
Daytime dysfunction (PSQI)	0.22	−0.09	−0.08	**0.48**	−0.02	0.14	0.78
Habitual sleep efficiency (PSQI)	0.13	−0.13	−0.01	**0.41**	−0.29	−0.02	0.34
Sleep disturbances (PSQI)	0.14	−0.12	−0.07	**0.39**	0.24	0.06	0.20
Sleep duration (PSQI)	0.01	−0.19	0.14	**0.38**	−0.04	−0.01	0.28
Factor 5: Task-oriented coping							
Task-oriented coping (CISS)	−0.07	0.49	−0.23	0.00	**0.50**	−0.03	0.25
Factor 6: Noticing							
Noticing (MAIA)	0.33	0.46	−0.04	0.14	−0.04	**0.55**	0.31

Note. CISS: Coping Inventory for Stressful Situations; MAIA: Multidimensional Assessment of Interoceptive Awareness; PERCI: Perth Emotion Regulation Competency Inventory; PSQI: Pittsburgh Sleep Quality Index; SMM-J: Stress Mindset Measure.

**Table 3:** Eigenvalues, proportion of variance explained, and cumulative proportion of variance explained for the 26 questionnaire components.

Component	Eigenvalue	% of variance	Cumulative %
1	6.4012	24.62	24.62
2	4.0648	15.634	40.254
3	2.4897	9.576	49.83
4	1.4492	5.574	55.404
5	1.3038	5.015	60.418
6	1.035	3.981	64.399
7	0.9635	3.706	68.105
8	0.8371	3.22	71.325
9	0.7592	2.92	74.245
10	0.6973	2.682	76.927
11	0.6627	2.549	79.476
12	0.6269	2.411	81.887
13	0.5404	2.078	83.965
14	0.5151	1.981	85.946
15	0.4812	1.851	87.797
16	0.4269	1.642	89.439
17	0.3896	1.498	90.937
18	0.3651	1.404	92.342
19	0.3321	1.277	93.619
20	0.2918	1.122	94.741
21	0.2833	1.089	95.831
22	0.2636	1.014	96.844
23	0.248	0.954	97.798
24	0.2108	0.811	98.609
25	0.1835	0.706	99.315
26	0.1781	0.685	100

**Table 4:** Factor correlation matrix.

Factor	1	2	3	4	5	6
Factor 1: Negative emotion regulation difficulty	–	0.01	0.07	0.07	−0.02	0.06
Factor 2: Interoception	0.01	–	−0.02	−0.03	0.05	0.03
Factor 3: Positive emotion regulation difficulty	0.07	−0.02	–	−0.04	−0.04	−0.02
Factor 4: Sleep problem	0.07	−0.03	−0.04	–	0.00	0.03
Factor 5: Task-oriented coping	−0.02	0.05	−0.04	0.00	–	0.00
Factor 6: Noticing	0.06	0.03	−0.02	0.03	0.00	–

Together, these six factors captured various individual factors relevant to emotional, behavioral, and physiological regulation. This factor structure served as the basis for subsequent moderation analyses that explored how these traits influenced the association between systemic inflammation and psychophysiological outcomes.

### 3.3 Moderating effects of individual factors on the Inflammation–Psychophysiological state relationship

Table 5, Table 6 present the descriptive statistics for inflammation and psychophysiological state measures. We conducted a hierarchical multiple regression analysis to examine whether the relationship between inflammatory markers and psychophysiological states was moderated by individual factors. Moderators included the six psychological traits identified via the factor analysis, and dependent variables included psycho-physiological state indices. All analyses were adjusted for age, sex, and BMI.

**Table 5:** Data of each inflammation markers.

Inflammation markers	Mean (pg/mL)	SD	N
CRP	−3.21	1.19	153
IL-6	−0.32	0.81	151
TNF-a	−0.79	0.36	155

Note. Inflammation data are log transformed. CRP: C-reactive protein; IL-6: interleukin-6; N: number of data analyzed; SD: standard deviation; TNF-α: tumor necrosis factor-alpha.

**Table 6:** Raw scores for the psychophysiological state questionnaires.

Questionnaire subscales	Mean	SD
**POMS 2**		
Anger-Hostility	43.61	8.51
Confusion-Bewilderment	46.92	10.28
Depression-Dejection	46.50	7.56
Fatigue-Inertia	43.74	9.29
Tension-Anxiety	45.14	10.44
Vigor-Activity	52.35	10.03
Friendliness	53.22	10.77
Total Mood Disturbance (TMD score)	44.23	9.07
**STAI**		
State anxiety	44.05	8.17
Trate anxiety	43.90	8.86
**CFSI**		
General fatigue	3.06	2.20
Chronic fatigue	2.14	2.35
Physical disorders	1.05	1.21
Depressive feeling	2.08	2.06
Feeling of anxiety	2.66	2.90
Decrease in vitality	2.29	2.60
Irritability	1.09	1.58
Unwillingness to work	2.48	2.94
**BDI-II**	7.52	6.59
**PSS**	25.23	9.23
**SRS-18**	10.05	10.64
**Stress VAS**	33.70	23.73

Note. Data are expressed as mean and SD. BDI-II: Beck Depression Inventory-II; CFSI: Cumulative Fatigue Symptoms Index; POMS 2: Profile of Mood States 2-A Short Form; PSS: Perceived Stress Scale; SD: standard deviation; SRS-18: Stress Response Scale-18; STAI: State-Trait Anxiety Inventory; Stress VAS: Stress Visual Analog Scale.

Multicollinearity was assessed via variance inflation factors (VIFs), all <2.0. Thus, multicollinearity was not a concern. Statistical power was deemed sufficient to detect moderate interaction effects (Cohen, 1988). Several significant main effects were observed in Model 1 (Table 7). Specifically, CRP demonstrated a significant positive association with physical complaints in individuals who reported high negative (*β* = 0.27, *p* = .001) and positive (*β* = 0.19, *p* = .020) emotion dysregulation. TNF-α was also associated with physical complaints among those with high levels of positive emotion dysregulation (*β* = 0.23, *p* = .009), poor sleep (*β* = 0.17, *p* = .046), and high task-oriented coping (*β* = 0.28, *p* = .002). However, this study focused on the interaction effects between inflammation and individual traits, rather than the main effects.

**Table 7:** Main effect of the multivariate analysis regarding associations between inflammation markers [pg/mL] and psychophysiological states for different individual factors (Model 1), adjusted for age and body mass index.

Variables	Inflammation markers	*β*	95 % Cl			*p* value	Adjusted R^2^	*n*
Sleep problem								
Chronic fatigue (CFSI)	CRP	0.15	−0.010	–	0.595	0.058	0.183	153
Vigor-Activity (POMS)	TNF-a	0.17	0.083	–	9.503	0.046	0.101	155
Positive emotion regulation difficulty								
Physical disorders (CFSI)	CRP	0.19	0.031	–	0.360	0.020	0.118	153
Physical disorders (CFSI)	TNF-a	0.23	0.195	–	1.320	0.009	0.113	155
Negative emotion regulation difficulty								
Physical disorders (CFSI)	CRP	0.27	0.117	–	0.434	0.001	0.173	153
Task-oriented coping								
Physical disorders (CFSI)	TNF-a	0.28	0.337	–	1.542	0.002	0.081	155

A total of 29 models demonstrated significant interaction effects (*p* < .05; Supplemental Material 3). Significant interactions were observed for models that involved negative and positive emotion regulation difficulties, sleep disturbance, interoceptive awareness, and task-oriented coping factors. In contrast, somatic awareness did not significantly interact with inflammatory markers.

Simple slope analyses were conducted to examine how the relationship between inflammation and psychophysiological states varied at high and low levels of each moderator for models with significant interactions. Of the 29 interaction models, 19 demonstrated at least one significant simple slope (Supplemental Material 4), which suggested that the nature of inflammation's impact on psychological states differed based on the individual factors. However, in 10 models, simple slopes were not significant, despite significant interactions. This suggested that the effects may be conditional or context-dependent, particularly in models that involved interoceptive awareness.

### 3.4 Key interaction effects

**Fig. 3:**
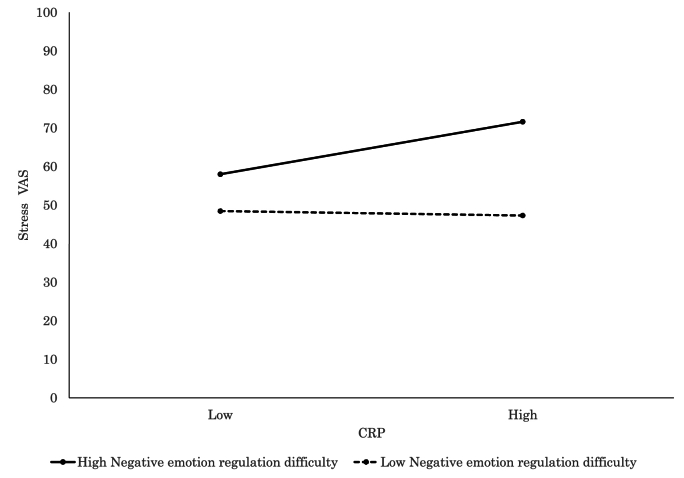
Significant associations between CRP and Stress VAS. CRP level was positively associated with stress for high Negative Emotion Regulation Difficulty, while no such association was observed for low Negative Emotion Regulation Difficulty.

**Fig. 4:**
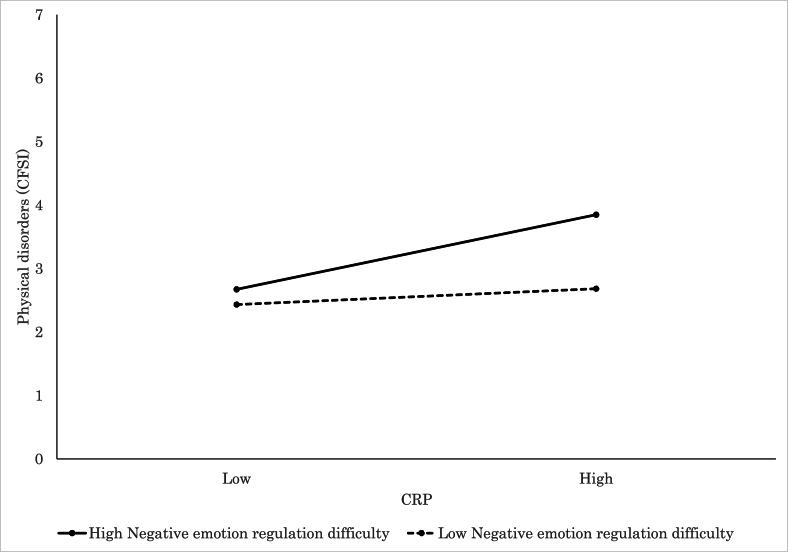
Significant associations between CRP and physiological disorder (CFSI). CRP level was positively associated with physiological disorder for high Negative Emotion Regulation Difficulty, while no such association was observed for low Negative Emotion Regulation Difficulty.

**Fig. 5:**
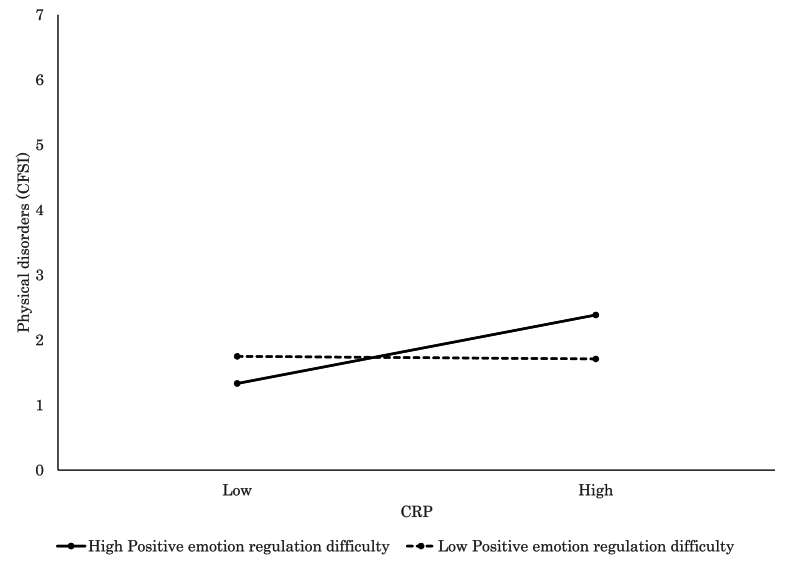
Significant associations between CRP and physiological disorder (CFSI). CRP level was positively associated with physiological disorder for high Positive Emotion Regulation Difficulty, while no such association was observed for low Positive Emotion Regulation Difficulty.

**Fig. 6:**
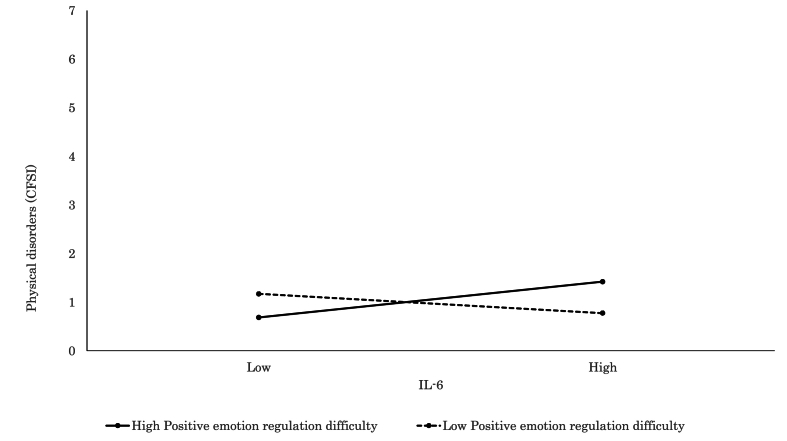
Significant associations between IL-6 and physiological disorder (CFSI). IL-6 level was positively associated with physiological disorder for high Positive Emotion Regulation Difficulty, while no such association was observed for low Positive Emotion Regulation Difficulty.

**Fig. 7:**
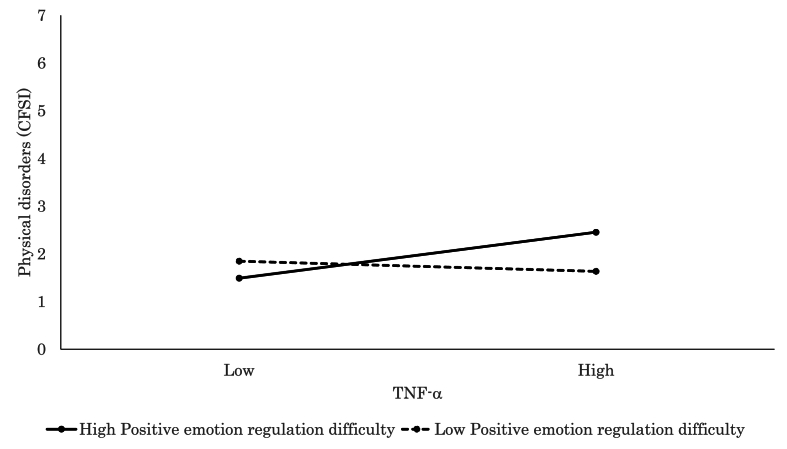
Significant associations between TNF-α and physiological disorder (CFSI). TNF-α level was positively associated with physiological disorder for high Positive Emotion Regulation Difficulty, while no such association was observed for low Positive Emotion Regulation Difficulty.

Detailed findings of the significant interaction models further elucidated how individual factors modulated the relationship between inflammatory markers and psychophysiological states. Elevated CRP was significantly associated with increased subjective stress (VAS; *β* = 0.28, *p* = .015, Fig. 3) and more severe physical complaints (CFSI; *β* = 0.46, *p* < .001, Fig. 4) among participants with high levels of negative emotion regulation difficulty, which suggested a pronounced vulnerability to inflammation-related distress. Similarly, CRP was associated with greater physical discomfort (CFSI; *β* = 0.42, *p* < .001, Fig. 5), heightened anxiety (CFIS; *β* = 0.21, *p* = .048), and more intense chronic fatigue symptoms (CFSI; *β* = 0.29, *p* = .007) among those with higher levels of positive emotion regulation difficulty. In parallel, IL-6 was also positively associated with feelings of fatigue and apathy (POMS 2; *β* = 0.22, *p* = .016), somatic symptoms (CFSI; *β* = 0.31, *p* = .001, Fig. 6), and diminished work motivation (CFSI; *β* = 0.19, *p* = .042), which indicated that increased inflammatory activity impaired both energy levels and functional engagement. TNF-α demonstrated similar associations with higher levels of physical complaints (CFSI; *β* = 0.47, *p* < .001, Fig. 7) and chronic fatigue (CFSI; *β* = 0.25, *p* = .040), which reinforced the robust link between inflammatory markers and bodily symptoms under conditions of emotion regulation difficulty.

Conversely, higher CRP levels were significantly associated with reduced depressive symptoms (BDI-II; *β* = 0.24, *p* = .045) and IL-6 levels were linked to lower psychosocial stress (SRS-18; *β* = 0.26, *p* = .047) among those with low levels of positive emotion regulation difficulty. These findings suggested that inflammation may not exacerbate, and may even buffer against, negative psychophysiological experiences under certain psychological conditions.

**Fig. 8:**
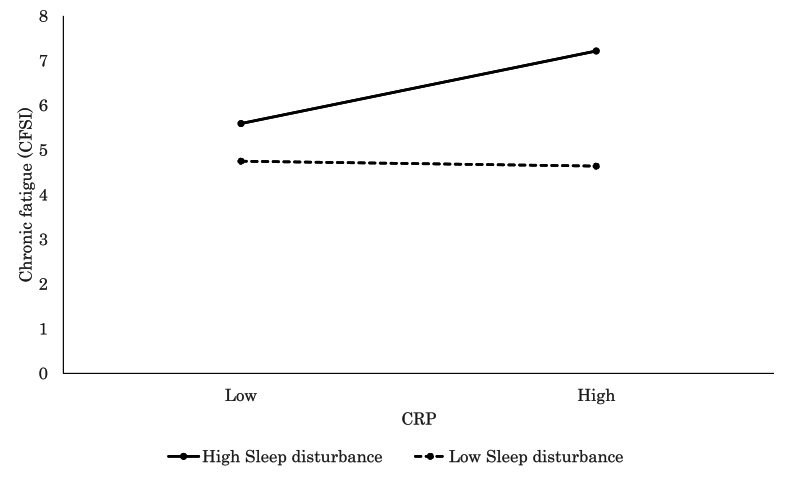
Significant associations between CRP and chronic fatigue (CFSI). CRP level was positively associated with chronic fatigue for high Sleep Disturbance, while no such association was observed for low Sleep Disturbance.

**Fig. 9:**
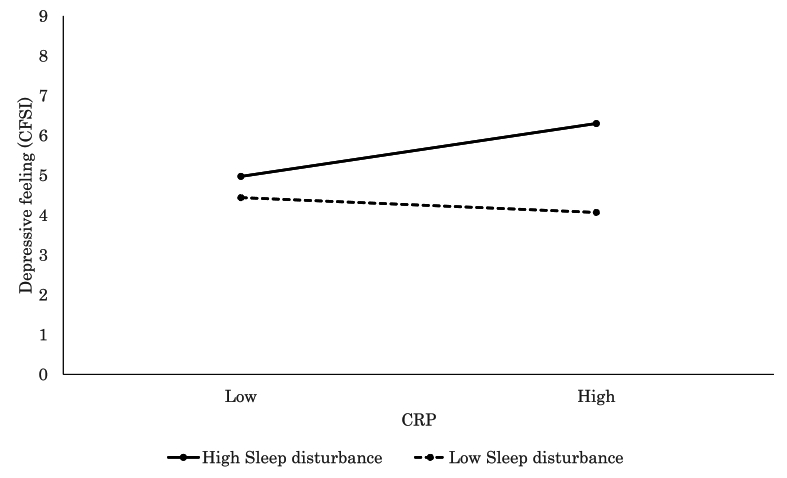
Significant associations between CRP and depressive feeling (CFSI). CRP level was positively associated with depressive feeling for high Sleep Disturbance, while no such association was observed for low Sleep Disturbance.

The moderating effect of sleep quality was similarly evident. CRP was significantly associated with more severe chronic fatigue (CFSI; *β* = 0.33, *p* = .004, Fig. 8), increased depressive symptoms (CFSI; *β* = 0.31, *p* = .007, Fig. 9), and greater anxiety (CFSI; *β* = 0.26, *p* = .017) among individuals with high levels of sleep disturbance. This suggested that poor sleep may sensitize individuals to the psycho-physiological impact of inflammation levels. In contrast, higher TNF-α levels were positively associated with increased vigor and vitality (POMS 2; *β* = 0.36, *p* = .003) among those with better sleep quality, which indicated a possible protective role of restorative sleep.

Although interaction terms that involved interoceptive awareness achieved statistical significance, subsequent simple slope analyses revealed no significant associations at either high or low levels (both *p* > .05). This suggested that its moderating effect, while statistically detectable, may be subtle or contingent on additional contextual factors.

Finally, task-oriented coping emerged as a nuanced moderator. CRP levels were significantly associated with lower depressive symptoms (BDI-II; *β* = 0.24, *p* = .042) among individuals with high task-oriented coping, which suggested an adaptive buffering role. However, higher TNF-α levels were also associated with increased physical complaints (CFSI; *β* = 0.45, *p* = .001) and elevated chronic fatigue (CFSI; *β* = 0.35, *p* = .015), which implied that even well-developed coping strategies may not uniformly mitigate the somatic effects of inflammation. Together, these results emphasize that individual factors function as critical moderators in the relationship between inflammation and psychophysiological states. Difficulties in emotion regulation and poor sleep quality exacerbate inflammation's adverse effects; conversely, individuals with better regulatory capacity or greater coping resources may experience attenuated, or even eliminated, effects. These findings provide compelling evidence for the inclusion of psychological moderators in psychoneuroimmunological models and may inform tailored interventions targeting vulnerable subgroups.

## 4 Discussion

This study investigated how individual factors, specifically interoception, emotion regulation, and sleep quality, moderated the relationship between inflammatory markers (CRP, IL-6, TNF-α, and IL-1β) and subjective psycho-physiological states in healthy individuals. Results revealed that difficulties in emotion regulation and poor sleep quality significantly intensified the association between elevated inflammation and adverse outcomes, which included heightened stress, depressive and anxiety symptoms, somatic complaints, and chronic fatigue. These results are consistent with those of prior research suggesting that individual differences influenced the impact of inflammation on psychological well-being. Thus, the findings support the hypotheses of the present study.

### 4.1 Interpretation of each modulating factor

Given the extensive number of questionnaire items related to individual factors, factor analysis was employed to consolidate them into six distinct factors: Negative Emotion Regulation Difficulty, Interoceptive Awareness, Positive Emotion Regulation Difficulty, Sleep Disturbance, Task-Oriented Coping, and Somatic Awareness. Subsequent analyses focused on how these factors moderated the relationship between inflammation and psychophysiological states.

Emotion regulation emerged as a critical moderator. Individuals with greater difficulty regulating negative emotions exhibited stronger associations between elevated CRP levels and increased stress and somatic symptoms (Slavich and Irwin, 2014). Similarly, difficulties in regulating positive emotions were associated with increased CRP and IL-6 levels and various symptoms, such as chronic fatigue, anxiety, and somatic complaints. These findings corroborated those of previous studies that indicated impaired emotion regulation exacerbated the psychological impact of inflammation.

Difficulties in regulating positive emotions may involve both the inability to downregulate heightened positive affect, potentially leading to excessive arousal, and the inability to upregulate or sustain positive affect, which is aligned with depressive tendencies. To clarify this factor, a post-hoc correlation analysis was conducted, with controls for age, sex, and BMI. Positive emotion regulation difficulty scores were significantly associated with irritability (CFSI), perceived stress (SRS-18), and anger/hostility (POMS) at *p* < .05, and demonstrated marginal associations with perceived stress (PSS) and depression (POMS) at *p* < .10. Results suggested that the factor primarily reflected a tendency toward excessive downregulation of positive emotions, which potentially reduced the ability to maintain positive mood. This aligned with the broader finding that emotion regulation difficulty promoted the persistence of negative states, which may enhance susceptibility to inflammation-related psychological burden (O'Connor et al., 2009). In contrast, individuals with stronger emotion regulation abilities demonstrated a lower association between inflammation and negative psychophysiological outcomes, which suggested a buffering effect. Previous studies separately linked adaptive strategies to better health and maladaptive ones to increased mental and physical burden (Appleton et al., 2013; Moriarity et al., 2023). However, this study integrated these findings and demonstrated that types and levels of emotion regulation moderated the psychological impact of inflammation even in healthy individuals.

Sleep quality also played a significant moderating role. Participants who reported poor sleep quality demonstrated stronger associations between elevated CRP levels and increased symptoms of depression, anxiety, and chronic fatigue (Irwin et al., 2016). These findings were consistent with those of prior research that linked sleep disturbances to heightened inflammation and worsened psychological outcomes. Sleep deprivation impaired emotion regulation and cognitive functions, leading to neural activity patterns similar to those observed in individuals with emotion regulation difficulties (Chee and Chuah, 2007; Tomaso et al., 2021). Such changes may be mediated by alterations in neuroendocrine and inflammatory responses (Haack et al., 2007; Irwin et al., 2008). Therefore, our findings suggest that changes in emotion regulation and cognitive functions due to poor sleep may serve as buffers in the relationship between inflammation and psychological states. This supports the notion that decreased emotion regulation abilities amplify the adverse effects of inflammation on psychological states. Notably, previous studies revealed that individuals with sleep disorders exhibited higher inflammation levels and positive correlations with depressive symptoms compared with healthy individuals (Yin et al., 2023). However, our findings indicated that similar relationships existed even among healthy individuals. These results suggest that poor sleep quality in healthy individuals may increase the risk of future disease onset, especially related to inflammation activity.

Interestingly, in individuals with effective emotion regulation and high sleep quality, elevated inflammation levels were associated with positive psycho-physiological states, such as reduced depression and enhanced vitality. Specifically, lower difficulties in positive emotion regulation were associated with higher CRP and IL-6 levels correlated with decreased depression scores and reduced stress responses, respectively. Additionally, lower poor sleep quality scores were associated with higher TNF-α levels correlated with increased vigor and vitality. These findings implied that inflammation did not invariably lead to worsened psychological states; rather, its effects varied based on individual factors, such as emotion regulation abilities and sleep quality. Since our study focused on healthy individuals, overall inflammation levels could be lower compared with those in patients with depression. Most importantly, these results suggest that the impact of inflammation on psycho-physiological states may be further influenced by psychological factors, such as emotion regulation and sleep quality, than by biological factors, such as inflammatory cytokines and neurotransmitter metabolism. Thus, interventions targeting psychological factors may offer new avenues for mitigating the psycho-physiological effects of inflammation and assessing future disease risk.

Regarding interoception, although interaction effects were observed, no significant main effects were observed. This suggested that interoceptive awareness may modulate the relationship between inflammation and psycho-physiological states through indirect or further complex mechanisms. Interoceptive awareness is involved in emotion regulation and stress response recognition (Critchley et al., 2004), and potentially interacts with other factors to exert modulatory effects. A study that examined the influence of inflammation levels and sensitivity to bodily information on depressive symptoms found that participants with low interoceptive awareness exhibited stronger associations between high inflammation levels and future depressive symptoms; conversely, no influence was observed in the relationship between inflammation levels and current depressive symptoms (Stephenson et al., 2024). This indicated that the effects of interoceptive awareness may be context-dependent and manifest over time (Gohm and Clore, 2000, 2002), which contributed to the worsening of depressive symptoms. Therefore, the role of interoceptive awareness in the relationship between inflammation and psychophysiological states may differ from that of the other two individual factors, necessitating further nuanced research designs and measurements to detect its effects.

### 4.2 Consideration of mechanisms underlying the interaction between modulating factors and inflammation

These findings support the notion that individual factors play a modulatory role in the relationship between inflammation and psychophysiological states, indicating that the impact of inflammation varies based on these factors. To deepen our understanding of these relationships, exploring the neural mechanisms underpinning the effects of individual factors is essential. Previous research has demonstrated that interoceptive awareness, emotion regulation, and sleep quality were all closely associated with specific brain regions involved in inflammation and emotional regulation.

Inflammation impaired the network functions of the PFC, crucial for emotion and behavior regulation, which contributed to deficits in emotion regulation and attention (Nusslock et al., 2019). Regarding sleep, studies that involved healthy individuals demonstrated that sleep deprivation leads to hyperactivity in the amygdala and reduced functional connectivity with the medial PFC in response to emotional stimuli (Yoo et al., 2007). This implied that sleep deprivation impaired top-down emotion regulation, which resulted in neural activity patterns similar to those observed in emotion regulation deficits (Tomaso et al., 2021). Other experimental studies that induced inflammation observed increased activity in the anterior insula and ACC, components of the interoceptive network, which supported the neural basis of the relationship between inflammation and interoceptive awareness (Kraynak et al., 2018; Savitz and Harrison, 2018). Furthermore, individuals with lower levels of anti-inflammatory gut bacteria exhibited reduced brain activity in regions associated with psychosocial stress and emotion regulation, such as the right premotor cortex, right dlPFC, right frontopolar cortex, and right inferior frontal gyrus/triangular part, which resulted in diminished stress responses (Yamaoka et al., 2022). These findings suggest that inflammation may weaken the PFC's regulation of the amygdala that lead to amplified negative emotions and stress and suppressed positive emotions, which deteriorated psychological states. Altogether, emotion regulation, sleep quality, and interoceptive awareness were associated with brain regions, such as the anterior insula, ACC, PFC, amygdala, and medial prefrontal cortex, which supports psycho-physiological functions, such as bodily state perception, emotional evaluation and selection, and inhibition. Investigating how changes in neural activity within these regions modulate the impact of inflammation on psychological states is crucial for advancing our understanding of the neurobiological basis of the inflammation-psychophysiological state relationship and reinforcing these findings.

### 4.3 Implications for prevention and intervention

These findings highlight the potential utility of individualized, further effective psychological and behavioral interventions in psychoneuroimmunology. Specifically, individuals with poor emotion regulation or low sleep quality exhibited a stronger association with elevated inflammatory markers and adverse mental and physical health outcomes, which included heightened perceived stress, somatic complaints, fatigue, depression, and anxiety. These results suggest personalized interventions tailored to individual psychological characteristics may be more effective in mitigating inflammation-related health risks, rather than standardized stress management and health promotion programs.

Psychological approaches, such as mindfulness-based or emotion regulation training, may help individuals with poor emotional control to downregulate their affective responses, which can reduce inflammation and improve mental well-being (Creswell et al., 2014). For individuals experiencing poor sleep quality, sleep hygiene education and cognitive behavioral therapy for insomnia (CBT-I) effectively enhanced both sleep and overall psycho-physiological health (Irwin, 2015).

Notably, all three moderating factors were functionally linked to brain regions implicated in affective regulation and bodily awareness, including the PFC, anterior insula, and ACC. This neurobiological insight suggests that interventions aimed at enhancing neural flexibility and integration (e.g., neurofeedback, meditation, regular physical activity) may also buffer against the psycho-physiological impact of inflammation. Moreover, Yamaoka et al. (2022) demonstrated that individuals with lower anti-inflammatory gut microbiota exhibited weaker PFC activation under psychosocial stress, which implied a potential gut–brain–immune axis. These findings suggest that interventions targeting gut health, such as probiotic or dietary modulation, might complement psychological interventions and serve as a multifaceted approach to preventing or alleviating inflammation-related mental and physical conditions.

### 4.4 Limitations and future directions

This study has several limitations. First, the cross-sectional design precludes any definitive conclusions regarding causality. Whether elevated inflammation leads to worsened mental states, or whether poor mental health contributes to increased inflammation remains unclear. Furthermore, considerable individual differences may exist in the reactivity and resolution of inflammatory responses to acute stressors, an aspect likely influenced by the moderators; however, these were not directly examined. Longitudinal experimental designs should clarify these temporal and causal pathways.

Second, some individuals with high inflammation but good emotion regulation or sleep quality did not exhibit any deterioration in subjective well-being. While this may indicate that regulatory traits buffer the adverse effects of inflammation, low-grade inflammation could go unnoticed in such individuals and progress toward chronic inflammation. Since this study was based on a single time-point, future longitudinal research should investigate how such individual traits may influence the persistence of inflammation and risk for developing related diseases.

Third, this study focused on three primary inflammatory markers: CRP, IL-6, and TNF-α. These reflect distinct physiological processes and may differ in their sensitivity to psychological factors. IL-6 and CRP were more strongly associated with psychosocial stress and behavioral changes and tended to vary more regarding psychological states (Marsland et al., 2006). Conversely, TNF-α reflected acute immune responses and local inflammation and demonstrated a more limited association with chronic psychological stress (Kiecolt-Glaser et al., 2015). IL-1β was also included but excluded from analyses due to a high proportion of values falling below the detection threshold, which suggested its limited utility in healthy adult populations.

Fourth, all the moderating variables (emotion regulation, sleep, and interoceptive awareness) were assessed through self-report measures, which may be susceptible to subjective bias. This is particularly relevant to interoception, where although interaction effects were observed, no significant simple slopes were found. This could be due to measurement sensitivity or the possibility that interoception influences health outcomes via more indirect or complex pathways. Future studies should incorporate objective measures, such as heartbeat detection tasks, to better capture interoceptive accuracy.

Fifth, as this study examined systemic inflammation alongside gut microbiota (not reported here), intake of foods and beverages known to influence the gut environment, including those rich in probiotics and prebiotics, were restricted during the experiment. Probiotics, such as Lactobacillus and Bifidobacterium, can promote anti-inflammatory cytokines, while prebiotics are fermented into short-chain fatty acids that exert anti-inflammatory effects (Bilal et al., 2022). Furthermore, many restricted foods overlap with anti-inflammatory food groups in EDIP analyses (Tabung et al., 2016), suggesting that effect of food on inflammation were minimized. Nevertheless, it remains possible that certain unaccounted-for foods may have influenced inflammatory activity. The use of the dietary inflammation index (Cavicchia et al., 2009; Hebert et al., 2019) might help clarify the effect of food in future studies. In addition, a small proportion of participants (∼4.5 %) reported consuming beer or red wine despite restrictions, and additional alcohol intake was also noted. Exploratory comparisons showed no significant differences in inflammatory markers, but small subgroup sizes limit certainty. In order to rule out the effect of the alcohol, future studies should adopt stricter exclusion criteria, such as abstinence for 48–72 h before blood sampling.

Lastly, the sample may not be representative of the general population, as it comprised a relatively homogeneous demographic regarding age and ethnicity. Cultural and contextual factors may moderate inflammation-related processes. Previous studies that compared Americans and Japanese individuals revealed that inflammation was associated with negative affect among Americans (Miyamoto et al., 2013). Future studies should explore how life stage, social background, and cultural context influence the moderating effects of individual traits on inflammation-related health outcomes.

## 5 Conclusion

This study demonstrated that individual differences in emotion regulation, sleep, and interoception significantly influenced the relationship between inflammation and subjective mental and physical well-being. In particular, individuals with poor emotional regulation or low sleep quality were more vulnerable to the negative effects of elevated inflammatory markers, such as CRP, IL-6, and TNF-α, associated with greater fatigue, somatic complaints, depression, and anxiety. Conversely, when emotion regulation and sleep were adequate, these associations were diminished or, in some cases, even reversed, which suggested that inflammation did not uniformly lead to adverse health outcomes; rather, it interacted dynamically with personal traits. Importantly, the impact of inflammation on psychophysiological states may be further influenced by psychological factors than by biological factors alone. Hence, interventions targeting both psychological and physiological aspects may offer new avenues for mitigating the psycho-physiological effects of inflammation and prevention of future risks**.**

## CRediT authorship contribution statement

**Kao Yamaoka:** Writing – original draft, Software, Resources, Methodology, Investigation, Formal analysis, Data curation, Conceptualization. **Yuri Ishii:** Supervision, Resources, Funding acquisition. **Yuri Terasawa:** Writing – review & editing, Supervision, Methodology, Conceptualization.

## Funding

This research was supported by internal research funding from FANCL Corporation (Yokohama, Japan). No external grants or public research funding were received.

## Declaration of competing interest

The authors declare that they have no known competing financial interests or personal relationships that could have appeared to influence the work reported in this paper.

## Data Availability

Data will be made available on request.

## Glossary

**Amygdala**: A brain region involved in emotional processing, particularly fear, threat detection, and the encoding of emotional memories
**Blood–Brain Barrier (BBB)**: A selective permeability barrier that protects the brain from potentially harmful substances in the bloodstream while allowing essential nutrients to pass
**BDI-II**: Beck Depression Inventory-II. A 21-item self-report questionnaire that assesses the severity of depressive symptoms
**Central Nervous System (CNS)**: Comprises the brain and spinal cord, responsible for processing and integrating information throughout the body
**CRP**: C-reactive protein. An acute-phase protein widely used as a biomarker of systemic inflammation
**Cumulative Fatigue Symptoms Index (CFSI)**: A self-report measure assessing chronic physical and psychological fatigue symptoms experienced in daily life.
**Dorsal Anterior Cingulate Cortex (dACC)**: A subregion of the anterior cingulate cortex involved in conflict monitoring, emotional regulation, and cognitive control
**Dorsolateral Prefrontal Cortex (dlPFC)**: A brain region implicated in executive functions such as working memory, planning, and top-down emotion regulation
**Efferent Vagus Nerve**: The branch of the vagus nerve that carries parasympathetic signals from the brain to peripheral organs, influencing inflammation and autonomic regulation
**Emotion Regulation**: The process of monitoring, evaluating, and modifying emotional reactions to meet situational or goal-related demands
**Exploratory Factor Analysis (EFA)**: A statistical method used to identify underlying latent factors or dimensions among observed variables
**Hypothalamic–Pituitary–Adrenal (HPA) Axis**: A neuroendocrine system that coordinates stress responses by regulating the secretion of cortisol and other stress hormones
**IL-1β**: Interleukin-1 beta. A pro-inflammatory cytokine involved in immune responses; excluded from analysis here due to low detectability
**IL-6**: Interleukin-6. A cytokine that plays a key role in acute and chronic inflammatory responses
**Interoception**: The perception and interpretation of internal bodily signals (e.g., heartbeat, respiration) that contribute to emotional experience and regulation
**MAIA**: Multidimensional Assessment of Interoceptive Awareness. A self-report instrument measuring awareness of internal bodily sensations
**Perceived Stress Scale (PSS)**: A self-report instrument that measures the degree to which individuals appraise situations in their lives as stressful
**PERCI**: Perth Emotion Regulation Competency Inventory. A scale assessing an individual's ability to regulate emotions adaptively
**POMS**: Profile of Mood States. A psychological rating scale that evaluates transient affective states such as tension, vigor, and fatigue
**Prefrontal Cortex (PFC)**: A brain area involved in executive function, emotion regulation, and decision-making
**PSQI**: Pittsburgh Sleep Quality Index. A questionnaire assessing subjective sleep quality over the past month
**Psychoneuroimmunology**: An interdisciplinary field that studies the interaction between psychological processes, the nervous system, and the immune system
**Sleep Disturbance**: Difficulties in initiating or maintaining sleep, or experiencing non-restorative sleep, leading to psychological or physiological consequences
**STAI**: State-Trait Anxiety Inventory. A 40-item inventory measuring state (situational) and trait (enduring) anxiety
**Sympathetic Nervous System**: A branch of the autonomic nervous system that mediates the body's 'fight-or-flight' response during stress
**TNF-α**: Tumor necrosis factor-alpha. A pro-inflammatory cytokine involved in systemic inflammation and immune regulation
**VAS (Stress)**: Visual Analog Scale for Stress. A single-item scale on which participants rate their current perceived stress level
