# Supplementary material for: Moderating effects of individual factors on the relationship between inflammation and psychophysiological states in healthy adults
Source: Brain Behav Immun Health. 2025 Nov 7;50:101135. doi: 10.1016/j.bbih.2025.101135 (PMC12666811; doi:10.1016/j.bbih.2025.101135)
Supplement: Multimedia component 1 [file mmc1.docx]

**Supplemental Material 1. Exclusion criteria.**

1. Individuals with symptoms of dementia or a prior diagnosis of dementia.
2. Individuals with a past or current diagnosis of alcohol-related disorders.
3. Individuals with depressive symptoms or a prior diagnosis of depression.
4. Individuals with a history of dysregulation of the hypothalamic–pituitary–adrenal axis.
5. Individuals undergoing hormone therapy or diagnosed by a physician with menopausal disorders.
6. Individuals with a history of gastrointestinal surgery (e.g., gastrectomy).
7. Individuals with periodontitis or currently under treatment for periodontitis.
8. Individuals who are pregnant or breastfeeding, or who may become pregnant during the study period.
9. Individuals engaged in night-shift or rotating-shift work that disrupts circadian rhythms, or those performing heavy physical labor such as carrying heavy loads.
10. Individuals who follow a vegetarian diet.
11. Current smokers or individuals who quit smoking within 12 months prior to obtaining consent.
12. Individuals receiving treatment for brain function or prescribed medications affecting brain function.
13. Individuals with diagnosed or self-reported sleep disorders (e.g., sleep apnea syndrome, restless legs syndrome, insomnia).
14. Individuals who habitually (≥ once per week) consume pharmaceuticals, health supplements, foods for specified health uses, foods with nutrient function claims, or foods with functional claims that may affect study outcomes (except those who can discontinue such intake from consent to the end of the study period).
15. Individuals unable to discontinue prebiotic/probiotic foods or other products containing active ingredients potentially influencing study outcomes during the 6 days from 5 days before to the day of study assessments.
16. Individuals currently under treatment for diarrhea or constipation.
17. Individuals who had taken antibiotics within 2 months prior to obtaining consent.
18. Individuals who had donated 200 mL or 400 mL of blood, or undergone component blood donation, within 3 months prior to obtaining consent.
19. Individuals who participated in another clinical trial (study) within 1 month prior to obtaining consent, or who plan to participate in another trial during the study period.
20. Individuals with severe current or past medical conditions requiring continuous medication, including neurological disorders, malignant tumors, immune disorders, diabetes, hepatic diseases (e.g., hepatitis), renal diseases, cardiac diseases, thyroid disorders, adrenal disorders, or other metabolic diseases.
21. Individuals deemed unable to comply with recording requirements of questionnaires.
22. Individuals judged inappropriate as participants by the principal investigator or study physician based on clinical test values, anthropometric data, or physical examination.
23. Any other individuals judged inappropriate as participants by the principal investigator or study physician.
