# Supplementary material for: Moderating effects of individual factors on the relationship between inflammation and psychophysiological states in healthy adults
Source: Brain Behav Immun Health. 2025 Nov 7;50:101135. doi: 10.1016/j.bbih.2025.101135 (PMC12666811; doi:10.1016/j.bbih.2025.101135)
Supplement: Multimedia component 2 [file mmc2.docx]

**Supplemental Material 2. Prohibited Foods During the Study Period. The following foods containing high in prebiotics and probiotics were prohibited from consumption during the study period (from the time of obtaining consent until the day of testing).**

- Japanese sweets containing red bean paste
- Sugarcane
- Cheese
- Cold potato salad
- Corn
- Fermented butter
- Beer
- Cold rice balls
- Okra, nameko mushrooms, mekabu (seaweed)
- Asparagus
- Amazake (sweet fermented rice drink)
- Potatoes
- Oatmeal
- Mushrooms (all varieties)
- Kimchi
- Cabbage
- Brown rice
- Konjac (konnyaku)
- Soy products
- Onion
- Pear
- Lactic acid bacteria beverages
- Garlic
- Nukazuke (rice bran pickles)
- Sticky/viscous foods (e.g., slimy-textured vegetables)
- Honey
- Cereals rich in fermentable dietary fiber
- Banana
- Prunes
- Broccoli
- Beans (all varieties)
- Melon
- Yogurt
- Rye and barley
- Seaweeds (all varieties)
- Agar
- Citrus fruits
- Sake lees and shio-koji (fermented rice seasoning)
- Red wine
- Natto (fermented soybeans)
- Miso
